# The Preoperative Inflammatory Status Affects the Clinical Outcome in Cardiac Surgery

**DOI:** 10.3390/antibiotics8040176

**Published:** 2019-10-05

**Authors:** Donato D’Agostino, Giangiuseppe Cappabianca, Crescenzia Rotunno, Francesca Castellaneta, Teresa Quagliara, Alessandro Carrozzo, Florinda Mastro, Ioannis Alexandros Charitos, Cesare Beghi, Domenico Paparella

**Affiliations:** 1 Department of Emergency and Organ Transplantations, Section of Cardiac Surgery, Consorziale Policlinico University Hospital, Bari-University of Bari, 70124 Bari, Italy; crescenzia.rotunno@uniba.it (C.R.); teresa.a.p.quagliara@gmail.com (T.Q.); alessandro_carrozzo@hotmail.it (A.C.); florinda.gmastro@gmail.com (F.M.); domenico.paparella@uniba.it (D.P.); 2 Department of Cardiac Surgery, “Circolo” Hospital, Insubria University, 21100 Varese, Italy; giangiuseppe.cappabianca@asst-settelaghi.it (G.C.);; 3 Department of Emergency/Urgency, Poisoning National Centre, “Riuniti” University Hospital, 71100 Foggia, Italy; fra_c@live.com (F.C.); alexanestesia@hotmail.com (I.A.C.)

**Keywords:** Inflammation, Inflammatory Status, C-Reactive Protein (CRP), Fibrinogen (FBG), Cardiac Surgery, Outcome, Clinical management, Infection risk, Sepsis, Cardio-Pulmonary Bypass (CBP)

## Abstract

Aims: There are many reasons for the increase in post-operative mortality and morbidity in patients undergoing surgery. In fact, an activated inflammatory state before cardiac surgery, can potentially worsen the patient’s prognosis and the effects of this preoperative inflammatory state in the medium-term remains unknown. Methods: There were 470 consecutive patients who underwent cardiac surgery, and were divided in three groups according to the median values of preoperative C-reactive protein (CRP) and fibrinogen (FBG): The first group was the low inflammatory status group (LIS) with 161 patients (CRP < 0.39 mg/dL and FBG < 366 mg/dL); the second was the medium inflammatory status group (MIS) with 150 patients (CRP < 0.39 mg/dL and FBG ≥ 366 mg/dL or CRP ≥ 0.39 mg/dL and FBG < 366 mg/dL,); and the third was the high inflammatory status group (HIS) with 159 patients (CRP ≥ 0.39 mg/dL and FBG ≥ 366 mg/dL,). Results: The parameters to be considered for the patients before surgery were similar between the three groups except, however, for age, left ventricular ejection fraction (LVEF) and the presence of arterial hypertension. The operative mortality was not significantly different between the groups (LIS = 2.5%, MIS = 6%, HIS = 6.9%, *p* = 0.16) while mortality for sepsis was significantly different (LIS = 0%, MIS = 1.3%, HIS = 3.7%, *p* = 0.03). The infections were more frequent in the HIS group (*p* = 0.0002). The HIS group resulted in an independent risk factor for infections (relative risk (RR) = 3.1, confidence interval (CI) = 1.2–7.9, *p* = 0.02). During the 48-months follow-up, survival was lower for the HIS patients. This HIS group (RR = 2.39, CI = 1.03–5.53, *p* = 0.05) and LVEF (RR = 0.96, CI = 0.92–0.99, *p* = 0.04) resulted in independent risk factors for mortality during the follow-up. Conclusions: The patients undergoing cardiac surgery with a preoperative highly activated inflammatory status are at a higher risk of post-operative infections. Furthermore, during the intermediate follow-up, the preoperative highly activated inflammatory status and LVEF resulted in independent risk factors for mortality.

## 1. Introduction

Heart disease, including coronary artery disease and heart valve disease, is often associated with underlying and an unrecognised inflammatory status. The inflammatory system is an extremely complex, inter-correlated mechanism, in which numerous stimuli, mediators and effectors intervene simultaneously. Therefore, a number of measurable inflammatory markers could be found elevated in the patients with heart disease. C-reactive protein (CRP) and fibrinogen (FBG) seem to be the most significantly associated with cardiovascular events [1]. In fact, CRP and FBG consistently predict new coronary events in patients with unstable angina and myocardial infarction [2,3,4,5], and mitochondrial oxidative stress has also been evidenced to be an independent risk factor for restenosis after percutaneous coronary intervention (PCI) [5,6,7,8].

Degenerative aortic stenosis has been recognized as an inflammatory disease with several histological analogies with coronary atherosclerosis [9]. Many authors have demonstrated that the patients with calcific aortic stenosis have increased CRP plasma levels which decrease after native valve replacement [10,11]. The high plasma levels of CRP are also common in patients with chronic rheumatic valve disease [12] and significantly predict the outcome after percutaneous balloon mitral valve commissurotomy [13].

Moreover, the patients with heart failure, regardless of the etiology, have a chronic activation of the inflammatory system which is probably supported by the renin-angiotensin-aldosterone system [12,14]. These patients have elevated plasma levels of CRP [15]. A relationship between elevated CRP levels and mortality, New York Heart Association (NYHA) class and hospitalization was also observed [15,16].

There are a few studies in the literature that analyse the effects of the preoperative inflammatory status on the prognosis of the patient undergoing cardiac surgery [17,18,19]. The authors observed that most of these show that the activated preoperative inflammatory state leads to a high incidence of infections after cardiac surgery. The effects of the pre-operative inflammatory status on the post cardiac surgery inflammatory reaction and on the mid-term outcome of the patients who have undergone surgical correction of their cardiac disease, remains to be demonstrated. The aim of this study is to evaluate the effects of the preoperative inflammatory status on a 48-month follow-up after cardiac surgery.

## 2. Patients and Methods

### 2.1. Study Design

**Figure 1:**
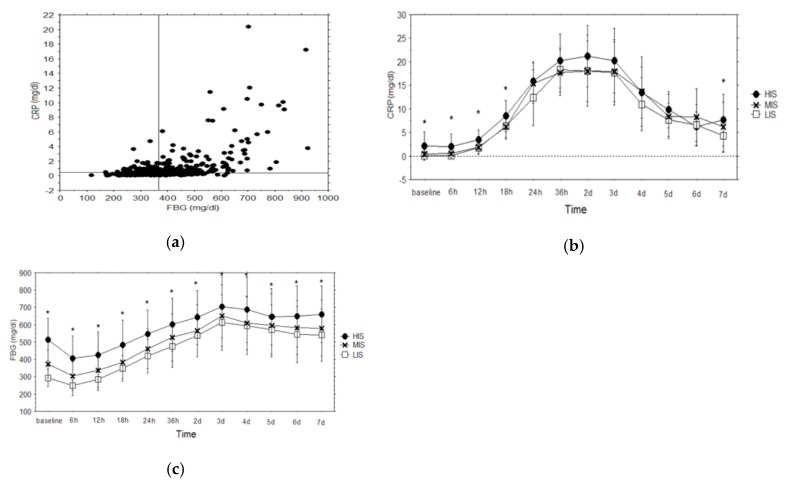
(**a**) **A** scattergram of preoperative C-reactive protein (CRP) and Fibrinogen (FBG) plasma levels of 470 patients who had undergone cardiac surgery. The two lines which cross the box represent the median values of CRP (0.39 mg/dL) and FBG (366 mg/dL). (**b**) Perioperative values of C-reactive protein (CRP) in the low inflammatory group (LIS), medium inflammatory group (MIS) and high inflammatory group (HIS). The asterisks mark differences with *p* ≤ 0.05. (**c**) Perioperative values of fibrinogen (FBG) in the low inflammatory group (LIS), medium inflammatory group (MIS) and high inflammatory group (HIS). The asterisks mark differences with *p* ≤ 0.05.

This study is based on a retrospective analysis of prospectively collected clinical data. Over a period of three years (February 2012–February 2015), 1789 patients who underwent cardiac surgery at the authors’ Institution were analysed. The patient candidates for cardiac operations were eligible in the study, except for the following exclusion criteria: emergency surgery, reoperations, the use of deep hypothermia presence of tumours or autoimmune disease and clinical signs of infections. This last condition was defined as the presence, at the time of hospital admission, of fever, or leukocytosis or of infective organ-related signs or symptoms (i.e.; productive cough, stranguria, sign of skin infections). Among the 578 patients in which preoperative CRP and fibrinogen values were available, 470 patients were suitable to be included in this study. The scattergram in Figure 1a shows the correlation between CRP and FBG baseline plasma levels (coefficient 0.57, *p* < 0.0001). With the goal of comparing the outcome of the patients with different preoperative activation of the inflammatory system, the median values of CRP (0.39 mg/dL) and FBG (366 mg/dL) were employed to divide the population in three groups: The low inflammatory status group (LIS), composed of 161 patients with CRP < 0.39 mg/dL and FBG < 366 mg/dL; the medium inflammatory status group (MIS) composed of 150 patients with CRP < 0.39 mg/dL and FBG ≥ 366 mg/dL or CRP ≥ 0.39 mg/dL and FBG < 366 mg/dL; the high inflammatory status group (HIS), composed of 159 patients with CRP ≥ 0.39 mg/dL and FBG ≥ 366 mg/dL. The clinical data were prospectively collected in the authors’ institutional electronic database by trained personnel.

The end points of this study related to the preoperative inflammatory status were twofold: First, to evaluate the effect on medium-term survival and their discharge from hospitalization for cardiac causes after cardiac surgery; second, to verify the effect on mortality and hospital morbidity.

### 2.2. Operation Techniques

The anesthetic management of the patients was with general anesthesia using lorazepam as a premedication and intravenous induction for intubation was done with fentanyl, midazolam and thiopental sodium. During the cardiac operation, general anesthesia was maintained by intravenous continuous controlled infusion of propofol. Median sternotomy was the surgical approach. Cardiac arrest was induced and maintained with cold-blooded anterograde cardioplegia and cardio-pulmonary bypass (CPB) was performed at moderate hypothermia (34 °C). Heparin (300 U/kg) was administered and intra-operative heparin monitoring was performed using the standard activated clotting time (ACT, Hemochron 8). The additional heparin bolus (5000 U) were given if the ACT value was below 400 seconds. Protamine was administered to reverse heparin (1 mg of protamine per milligram of total heparin given before and during CPB). The off pump coronary revascularizations were carried out with the use of Lima stitch and intracoronary shunts in all patients. Anesthesia and anticoagulation management was not different to the one used for the patients who had undergone CPB. In all the patients who had undergone valve replacement, a mechanical bi-leaflet prosthesis was implanted.

### 2.3. Definitions

Unstable angina was defined as on-going refractory angina that requires the use of intravenous nitrate therapy for control. The left ventricular ejection fraction (LVEF) was obtained in all the patients by left ventriculogram. Post-operative mortality was defined as the death, regardless of the cause in the same hospital admission of the patients after surgery. Cardiac death has been defined as a death due solely to heart damage, including sudden death as an unexpected death due to cardiac causes occurring immediately after the onset of symptoms (within an hour) or even without the onset of symptoms. The post-operative presence of low cardiac output syndrome (LOS) was defined as the reduction of cardiac output and the application in support of cardiac function and cardiac output of inotropic and post-load reducing agents or through mechanical circulatory support with an intra-aortic balloon pump for more than 30 minutes in intensive care unit (ICU). The goal was to maintain systolic blood pressure (SP) > 90 mm Hg, the mean arterial pressure (MAP) > 60 mm Hg) or the cardiac index (CI) > 2.2 L/min/m^2^, despite adequate volume resuscitation. According to anesthesiologists’ protocols, extubation criteria were: hemodynamic stability, the absence of surgical bleeding, fully re-warming, awakeness, optimal blood gases with FIO_2_ ≤ 0.3 and without the need for mechanical assistance. Sepsis was defined according to its current classification by at least two of the following clinical criteria: body temperature < 36 °C or > 38 °C; heart rate > 90 beats/minute; respiratory rate > 20 breaths/minute; PCO_2_ < 32 mm Hg; white blood cells > 12000 or < 4000/L [20].

### 2.4. Biochemistry

The blood samples were collected pre-operatively on the morning of the operation and at 1, 6, 12, 24, 36 hours after the end of the operations and every day from the second to the seventh post-operative day. The high-sensitivity C-reactive protein (hsCRP) measurements were detected based on the immunoassay technique through Flex^®^ reagent cartridge, Dade Behring Inc.; Newark, DE, and FBG was measured with the Clauss clotting time method (Biopool Fibrinogen Assay Kit, Biopool International, Ventura, CA, USA).

### 2.5. Follow-up

After being discharged from the hospital, the patients were evaluated periodically at the follow-up by going to the authors’ Institute or by contacting their general practitioner by phone call or by the medical staff. The follow-up was 94.8% complete (423/446). The maximum and mean follow-up were 48.6 and 19.6 ± 11 months.

### 2.6. Statistical Analyses

The continuous variables were presented as the mean ± standard deviation and were compared with an analysis of variance (ANOVA). The categorical variables were presented as absolute numbers and percentages and were compared using the χ^2^ test. The repeated ANOVA measures were employed to evaluate the differences in postoperative plasma levels of CRP and FBG between the three groups. The differences between the study groups were considered statistically significant when *p* ≤ 0.05. The outcome variables were significantly different for the univariate analysis, and underwent multivariate analysis by logistic regression, matching the preoperative inflammatory status versus the pre and intra-operative variables which had results different from the comparison of the three groups. For the outcome, the variables were independently influenced by the inflammatory status. The receiver operating characteristic curves (ROC curves) were used to establish the best cut-off of CRP and FBG. The Kaplan and Mayer curves were employed to evaluate survival and freedom from hospitalization for cardiac events. The curves of the three groups were compared by the log rank test. If the log rank test was significant, the Cox model was applied to verify the independent effect of the group on the outcomes, compared to the other significantly different variables. The statistical analyses were performed using the Stat-View Statistical Software Package (SAS Institute Inc. Cary, NC, USA) and Number Cruncher Statistical System (NCSS, Kaysville, Utah, USA).

## 3. Results

### 3.1. Laboratory Results

The repeated ANOVA measures showed that the patients with preoperative highly activated inflammatory status have significantly higher plasma levels of CRP during the postoperative period (f-ratio = 4.28, *p* = 0.01). CRP mean the preoperative values (Figure 1b) were: 0.18 ± 0.09 mg/dL for the LIS group, 0.54 ± 0.59 mg/dL for the MIS group and 2.2 ± 3.02 for the HIS group (*p* < 0.0001). The CRP postoperative peak values were observed 48 hours after the operation (LIS = 18.1 ± 6.4 mg/dL, MIS = 18.1 ± 7.5 mg/dL, HIS 21.2 ± 6.4 mg/dL, *p* = 0.11). In the seventh post-operative day, the patients of the HIS group still had significantly higher CRP plasma levels (LIS = 4.3 ± 3.4 mg/dL, MIS = 6.2 ± 5.2 mg/dL HIS = 7.7 ± 5.4 mg/dL, *p* = 0.01). The fibrinogen mean preoperative values (Figure 1c) were: 292.4 ± 49.3 mg/dL in the LIS group, 371.1 ± 80.6 mg/dL in the MIS group and 513.3 ± 123.2 mg/dL in the HIS group (*p* < 0.0001). Postoperatively, after an initial drop probably caused by hemodilution, the fibrinogen peak values were observed in the third post-operative day (LIS = 612 ± 159.7 mg/dL, MIS = 650.3 ± 177.8 mg/dL, HIS = 702.2 ± 178.4 mg/dL *p* = 0.0002). In the seventh post-operative day, the HIS group still had significantly higher FBG plasma levels (LIS = 538.6 ± 150.2 mg/dL, MIS = 579.6 ± 160.1 mg/dL, HIS 659 ± 164.1 mg/dL *p* < 0.0001). The repeated ANOVA measures confirmed that the patients with a preoperative inflammatory status have significantly higher fibrinogen plasma levels in the postoperative period (f-ratio = 2.69, *p* < 0.0001).

### 3.2. Clinical Results

The preoperative clinical status was comparable among the groups except for age, preoperative left ventricular ejection fraction and the incidence of hypertension (Table 1). The preoperative assumption of statins was similar in the three groups. The types of surgical procedures performed in the three groups were equally distributed but the CPB time was longer in the patients of the HIS group (Table 2). The off-pump procedures were also similarly distributed in the three groups. Overall, operative mortality was 5.1% (24/470), 2.5% in the LIS group, 6% in the MIS 6% and 6.9% in the HIS group without a statistically significant difference (*p* = 0.16). The causes of death are listed in Table 3. The patients in the HIS group had a significantly higher incidence of death caused by sepsis. The univariate analysis of post-operative morbidity is shown in Table 2: A preoperative highly activated inflammatory status is associated with a significantly higher incidence of mechanical ventilation >24 h, the need of dialysis/ultrafiltration, sepsis, sternal wound and overall infections. In the multivariate analysis, the preoperative high inflammatory status (HIS) was the only independent risk factor for sternal wound infections (risk ratio (RR) = 3.5, CI = 1.04–11.8, *p* = 0.04) and overall infections (RR = 3.1, CI = 1.2–7.9, *p* = 0.02). The ROC curves evidenced that the best cut-off values to predict sternal wound infections were 0.66 mg/dL for CRP (sensitivity 68.7%, specificity 69.8%, area under the curve (AUC) = 64.7%) and 367.9 mg/dL for FBG (sensitivity 75%, specificity 52.5%, AUC = 64.6%). The best cut-off values to predict overall infections were 0.66 mg/dL for CRP (sensitivity 62%, specificity 70.7%, (AUC) = 64.6%) and 367.9 mg/dL for FBG (sensitivity 72%, specificity 53.3%, AUC = 64.3%).

### 3.3. Follow-up

**Figure 2:**
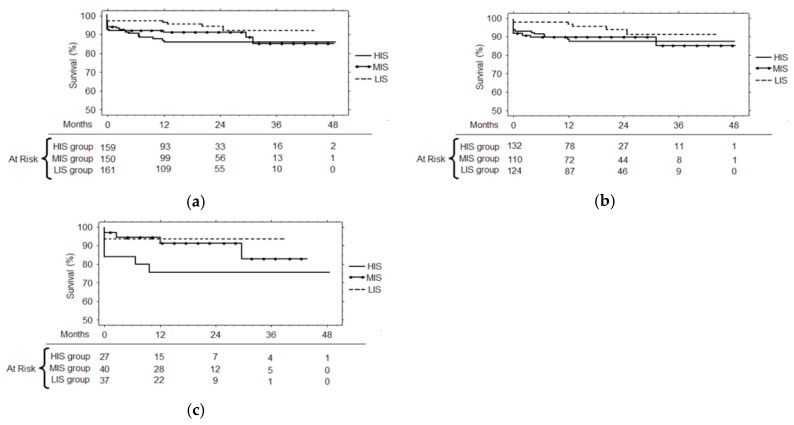
(**a**) The 48-months survival for patients of the LIS, MIS and HIS group. (**b**) Subgroup of patients undergone coronary artery bypass grafting (CABG): The 48-months survival for patients of the LIS, MIS and HIS group. (**c**) The subgroup of patients undergone cardiac surgery other than CABG: The 48-months survival for patients of the LIS, MIS and HIS group.

**Figure 3:**
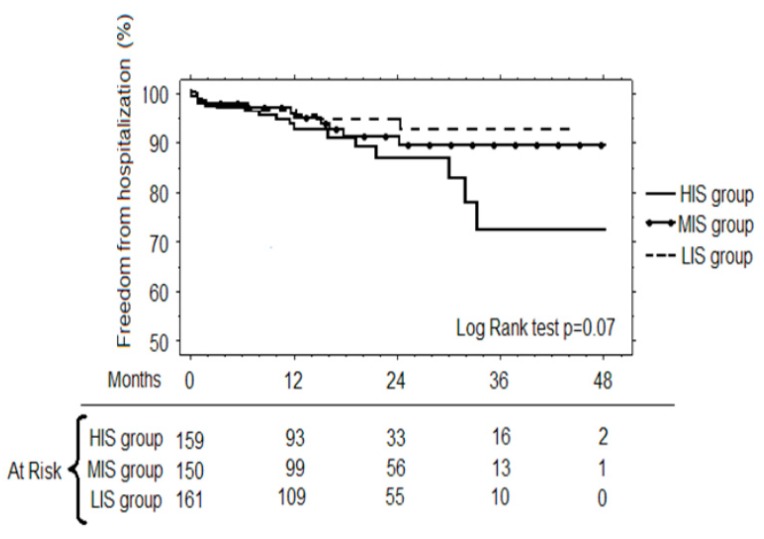
The 48-months freedom from hospitalization for cardiac events for patients of the LIS, MIS and HIS group.

At forty-eight months survival (Figure 2a) among the three groups of the patients, it was demonstrated that the best survival was that of the LIS (91.9 ± 3.1%) compared to the MIS (84.3 ± 4.8, *p* = 0.13) and the HIS (85.7 ± 3.0%, *p* = 0.01). Furthermore, the Cox regression revealed for the HIS group, the risk ratio for infections (RR = 2.39, CI = 1.03–5.53, *p* = 0.05) and the LVEF (RR = 0.96, CI = 0.92–0.99, *p* = 0.04) were independent risk factors for mortality during 48-months follow-up. The ROC curves for survival evidenced that the best CRP cut-off value was 0.5 mg/dl (sensitivity 59.1%, specificity 59.6%, AUC = 60.2%), the best FBG cut-off value was 321 mg/dL (sensitivity = 90.9%, specificity = 34.8% AUC = 63%), and the best cut-off LVEF was 42% (sensitivity 47.3%, specificity 78.4%, AUC = 61.3%). Furthermore, in the subgroup of the patients who had undergone coronary artery bypass grafting (CABG) (Figure 2b), survival was better for the LIS group (91.5 ± 3.7%) compared to the MIS group (85.4 ± 5.4%, *p* = 0.16) and the HIS group patients (87.7 ± 3.1%, *p* = 0.06). In the subgroup of the patients who had undergone cardiac surgery other than CABG (Figure 2c), survival was 93.8 ± 4.3% for the LIS group, 82.9 ± 9.1% for the MIS group (*p* = 0.69 compared to LIS group) and 75.6 ± 8.7% for the HIS group (*p* = 0.06 compared to LIS group). The freedom from hospitalization for cardiac events (Figure 3) was higher for the LIS group (92.2 ± 2.9%) compared to the MIS group (88.6 ± 3.4%, *p* = 0.38) and the HIS group patients (72.2 ± 8.4%, *p* = 0.07).

## 4. Discussion

CRP is an acute-phase protein mainly produced by the liver in response to interleukin 6 (IL-6). It is a pentameric protein composed of five identical units that are implicated in the synthesis of interleukin 1 and tumour necrosis factor, in the modulation of macrophage activity and in the activation of the complement system [21]. FBG is the substrate for the fibrin production, participates in platelet aggregation and acts as acute phase protein, increasing during inflammatory responses. Combining CRP and FBG values, three different preoperative inflammatory status levels were defined in this study. It is believed that the association of 2 reliable markers allowed the identification of a group of patients with an extremely activated preoperative inflammatory state (HIS group). This study would not have had the same selection by using a single inflammatory marker. The degree of the preoperative inflammatory status in the patients undergoing cardiac surgery can be influenced by several factors. The development of atherosclerotic plaques depends considerably on the inflammatory cascade and therefore, this complication appears to be the inflammatory response to endothelium lesions due to the various causes of risk such as hypertension, smoking, diabetes and hypercholesterolemia [1]. In this study, the patients with a high inflammatory status had an elevated incidence of hypertension, while smoking history, diabetes mellitus and hypercholesterolemia were similarly distributed. The patients of the HIS group had a higher incidence of unstable angina and recent myocardial infarction, although the difference was not statistically significant. Other reports evidenced significantly higher levels of CRP and IL-6 in the patients with unstable angina and myocardial infarction, supporting the connection between acute coronary syndromes and inflammatory status [1,2,4]. In this study, the patients with a highly activated preoperative inflammatory status (HIS) had a significantly lower LVEF, and a correlation between left ventricular dysfunction and CRP has already been reported [14,15,16]. The patients with calcific aortic stenosis and chronic rheumatic valve disease were similarly distributed in the three groups. This finding is in contrast with other studies [9,10,11] that reported high preoperative CRP plasma levels in these two kinds of diseases.

Following surgery, the CRP plasma levels were observed to increase, reaching the peak in the second post-operative day. This finding is consistent with those reported by Gaudino and co-workers [22]. Before the discharge home, the HIS group patients had still higher levels of CRP. Compared to CRP, FBG remains higher until discharge, acting as a late acute phase protein. This study shows that the preoperative inflammatory status significantly influences the magnitude of the inflammatory reaction provoked by the operation. Therefore, the preoperative inflammatory status could be considered a risk factor for post-operative inflammatory reaction, magnifying the effects of the intraoperative inflammatory stressors (cardiopulmonary bypass, ischemia, ischemia-reperfusion injury, endotoxin release and surgical trauma). The development of postoperative complications such as myocardial dysfunction, respiratory failure, renal and neurologic dysfunction, bleeding, altered liver function, and ultimately, multiple organ failure may relate to the degree of the postoperative inflammatory reaction. The possibility to predict the magnitude of this reaction may facilitate the adoption of different pharmacological or technical strategies (administration of glucocorticoids, protease inhibitors, antioxidants, use of coated circuits, pulsatile flow during CPB, the use of techniques aimed at reducing oxidative stress, etc.), able to minimize the post-operative inflammatory reaction in patients at risk [23,24,25,26,27,28,29,30,31,32,33,34,35,36].

In this study, the patients with a preoperative highly activated inflammatory status (HIS) had a higher, but not statistically significant hospital mortality. The data in this study confirm that an increased preoperative inflammatory state facilitates postoperative infective complications. Biancari and colleagues [17] reported that approximately 764 patients who had undergone CABG, preoperative PCR >1 mg/dL was recognized as an independent risk factor for operative mortality (RR 6.97, CI = 1.44–33.42, *p* = 0.01). In this study, although the HIS group patients had worse preoperative clinical conditions, an increased rate of operative mortality for cardiac causes was not observed, while the mortality for sepsis was significantly influenced by the preoperative inflammatory status. The multivariate analysis showed that the preoperative inflammatory status was the only independent risk factor for post-operative infections. A previous report by Boeken [18] regarding 100 patients undergone cardiac surgery with cardiopulmonary bypass evidenced that patients with preoperative CRP > 0.5 mg/dL had higher incidence of septic complications, need of inotropic support and a significantly longer duration of mechanical ventilation and intensive care unit (ICU) stay. Accordingly, Fransen [19] evidenced that a CRP > 0.8 mg/dL was an independent risk factor for post-operative infections (RR = 2.7, CI = 1.7–4.3, *p* < 0.0001) [22]. The two hypotheses of why the higher rate of postoperative infectious complications occur in patients with a high preoperative inflammatory status can be observed. The first is based on the observation that bacterial growth increases in the presence of high concentrations of pro-inflammatory mediators with related mortality to the degree of the spread and duration of the inflammatory response of the host [37,38]. The second, as demonstrated by Meduri and colleagues, is that in patients with an acute respiratory distress syndrome, the development of a pneumonia is associated with the ventilator that can cause an increased mortality risk that it is also related to the degree of the spread and duration of the inflammatory response of the host [39,40].

In this study, the HIS group patients had a higher incidence of mechanical ventilation (>24 h). It has been also demonstrated that the grade of postoperative inflammation after cardiopulmonary bypass, measured through CRP and migration inhibitory factor (MIF), is strongly connected with degree of pulmonary dysfunction. The MIF levels measured six hours after CPB were inversely related to the postoperative PaO_2_/FiO_2_ ratio and were directly related to the duration of mechanical ventilation [41].

The original finding of this study was that the patients with a high preoperative inflammatory status (HIS) during a 48-month follow-up, had significantly lower survival compared to the patients with a low preoperative inflammatory status (LIS). Moreover, a high inflammatory status and LVEF were independent risk factors for survival. Similar trends were demonstrated in the subgroups of the patients who had undergone CABG and cardiac surgery other than CABG.

The CRP power to predict the adverse cardiac events has been better defined. A large prospective long-term study based on 27,939 healthy women showed that healthy patients with CRP > 0.41 mg/dL (higher quintile) have significantly lower event-free survival from cardiovascular events in comparison with the patients with CRP < 0.04 (lower quintile) [42]. A sub-analysis on the same population evidenced the strong prognostic value of very high levels of CRP (>2 mg/dL) on the incidence of cardiovascular events (adjusted RR 3.1) [43]. Several prospective studies evidenced that high pre-procedural levels of CRP and FBG significantly affect the long-term survival, freedom from major cardiac events and restenosis rates after PCI [44,45,46,47]. In the patients with coronary artery disease, the individual’s reactivity to inflammatory stimuli plays an important pathophysiologic role in the progression of atherosclerotic plaques. The presence of a high-grade inflammatory status before CABG could be considered a marker of activity of atherosclerotic disease which can affect the long-term outcome.

Unfortunately, the mechanisms by which CRP and fibrinogen affect the long-term outcomes in the patients who have undergone cardiac surgery different than CABG remain less clear and hypothetical. Krasuski and co-workers evidenced the prognostic value of CRP after percutaneous balloon mitral valve commissurotomy [12]. Moreover, it has been clarified that the patients with either ischemic and non-ischemic heart failure have a chronic activation of the inflammatory network [13,14] and other reports have evidenced that high CRP plasma levels were independent risk factors for hospitalization in the patients with heart failure and were associated with death during the 18-months follow-up [15,16].

## 5. Conclusions

This study highlighted the presence of an association between a preoperative inflammatory status, defined by CPR and fibrinogen, both above the median values (CRP ≥ 0.39 mg/dL and FBG ≥ 366 mg/dL,), and the occurrence of postoperative infections, particularly regarding the sternal wound. Moreover, a trend toward statistical significance was found for hospital mortality. This finding is supported by the literature [42,43,44,45,46,47,48,49,50,51,52].

The patients receiving the operation with an activated inflammatory system showed an increased inflammatory reaction to cardiac surgery. In the mid-term, the patients with preoperative highly activated inflammatory status had a poor prognosis despite surgical correction of their cardiac disease. This finding seemed to be confirmed either in patients who had undergone CABG and in patients who had undergone cardiac surgery other than CABG. The relationship between the inflammatory status, activity of atherosclerotic processes and left ventricular dysfunction could explain these results. On the other hand, the preoperative inflammatory state is associated with many other events (left ventricular dysfunction, hypertension, the need for other surgical procedures etc.), which can potentially increase the risk for postoperative infection and decrease mid-term survival. Moreover, the co-existing systemic conditions, (i.e.; cancers, recent surgery, diabetes, etc.) can often worsen the outcome of cardiac surgery, especially if they are associated with chronic inflammation mostly related to periodontitis or dental decays [53,54,55,56,57,58,59]. This is well known from the mid-1950s, so prophylactic dental hygiene is actually prescribed to patients that must have cardiac surgery [60,61,62]. Then, a correct management of the patients that must have cardiac surgery is mandatory and must include traditional therapies [63,64,65,66,67,68] and, prospectively, innovative tools and procedures [69,70,71,72]. If sepsis arises in the patients who are candidates for cardiac surgery, or have undergone a rapid and correct diagnosis, this should be done from the time of admission to the Emergency Departments, in order to reduce the risk of death [73,74,75].

The limits of the study are the lack of a prospective design, the limited number of patients for each subset of diseases and the absence of data regarding the statins assumption during the follow-up. Another limit is that post-operative factors as a long duration of stay in the intensive care unit, a higher incidence of mechanical ventilation or post-operative dialysis, etc. may affect the inflammatory status of the patient and could play a role, influencing the outcome.

Future trials are needed in order to ascertain a mid-term protective role of anti-inflammatory agents in the patients who have undergone cardiac surgery with an elevated inflammatory status.

## Figures and Tables

**Table 1:** Preoperative characteristics.

	LIS	MIS	HIS	*p*
Patients	161	150	159	
Age (years)	63.1 ± 10.2	65.5 ± 9.6	66.2 ± 8.8	0.01
Male	126 (78.3%)	104 (69.3%)	116 (73%)	0.19
BSA (m^2^)	1.79 ± 0.18	1.76 ± 0.16	1.78 ± 0.16	0.46
Unstable Angina	19 (11.8%)	23 (15.3%)	25 (15.7%)	0.54
MI < 21 days	31 (19.3%)	29 (19.3%)	41 (25.8%)	0.26
Calcific Aortic Stenosis	12 (7.4%)	15 (10%)	9 (5.6%)	0.35
Rheumatic valvular disease	8 (4.9%)	7 (4.7%)	4 (2.5%)	0.48
LVEF (%)	49.2 ± 8.8	49.9 ± 9.9	46.8 ± 10.6	0.02
Hypertension	88 (54.7%)	94 (62.7%)	115 (72.3%)	0.005
Diabetes	49 (30.4%)	41 (27.3%)	50 (31.4%)	0.71
Hypercholesterolemia	75 (46.6%)	83 (55.3%)	89 (56%)	0.17
Statins therapy	55 (34.2%)	61 (40.6)	67 (42.1)	0.29
Smoking history	37 (23%)	34 (22.7%)	52 (32.7%)	0.07
COPD	39 (24.2%)	37 (24.7%)	46 (28.9%)	0.57
C.A. Stenosis > 60%	7 (4.3%)	9 (6%)	13 (8.2%)	0.36
CVA < 21 days	1 (0.6%)	1 (0.7%)	4 (2.5%)	0.23
Preop. cTnI (ng/mL)	0.11 ± 0.42	0.21 ± 1.2	0.40 ± 1.43	0.15

BSA = body surface area; MI = myocardial infarction; LVEF = left ventricular ejection fraction; COPD = chronic obstructive pulmonary disease; C.A. = carotid artery; CVA = cerebrovascular accident; Preop cTnI = preoperative cardiac Troponin I.

**Table 2:** Operative variables.

	LIS	MIS	HIS	*p*
Patients	161	150	159	
CABG	79 (49.1%)	72 (48%)	81 (50.9%)	0.87
OPCAB	38 (23.6%)	30 (20%)	44 (27.7%)	0.28
Aortic Root surgery	3 (1.9%)	3 (2%)	6 (3.8%)	0.48
AVR	12 (7.5%)	17 (11.3%)	9 (5.7%)	0.17
Mitral replacement/repair	7 (4.3%)	14 (9.3%)	10 (6.3%)	0.2
Combined procedures	16 (9.9%)	13 (8.7%)	8 (5%)	0.24
Other procedures	6 (3.7%)	1 (0.7%)	1 (0.6%)	0.06
CPB duration (min)	105 ± 28	114 ± 44	120 ± 34	0.006
X-Clamp duration (min)	58 ± 22	63 ± 23	62 ± 23	0.3

OPCAB = off-pump coronary artery bypass; AVR = aortic valve replacement; CPB = cardiopulmonary bypass.

**Table 3:** Post-operative mortality (M 1) and morbidity (M 2), related to pre-operative inflammatory status.

	LIS	MIS	HIS	*p*
**M 1**				
Patients	161	150	159	
Operative Mortality	4 (2.5%)	9 (6%)	11 (6.9%)	0.16
Causes of death				
Cardiac death	4 (2.5%)	5 (3.3%)	3 (1.8%)	0.72
Sepsis	0	2 (1.3%)	6 (3.7%)	0.03
Stroke	0	1 (0.7%)	1 (0.6%)	0.59
Bleeding	0	1 (0.7%)	1 (0.6%)	0.59
**M 2**				
ICU stay (h)	46.9 ± 28.5	62.1 ± 78.9	65.6 ± 101.2	0.07
Overall stay (days)	8.1 ± 4.9	9.9 ± 8.2	9.7 ± 71.8	0.06
Mechanical ventilation > 24 h	13 (8.1%)	26 (17.3%)	32 (20.1%)	0.007
Reintubation	3 (1.9%)	8 (5.3%)	8 (5%)	0.22
LOS	19 (11.8%)	26 (17.3%)	26 (16.4%)	0.34
IABP	4 (2.5%)	9 (6%)	7 (4.4%)	0.31
cTnI peak (ng/mL)	15.9 ± 35.2	15.5 ± 19.6	19.5 ± 49.5	0.56
Dialysis/Ultrafiltration	0	4 (2.7%)	7 (4.4%)	0.03
AF onset	43 (26.7%)	41 (27.3%)	41 (25.8%)	0.95
CVA	0	4 (2.7%)	4 (2.5%)	0.11
Sepsis	0	5 (3.3%)	6 (3.8%)	0.05
Sternal wound infection	5 (3.1%)	7 (4.7%)	20 (12.6%)	0.002
Overall infections	9 (5.6%)	11 (7.3%)	30 (18.9%)	0.0002
Blood loss (ml)	840.4 ± 432.4	745.4 ± 406.7	776.2 ± 500.1	0.17
Reopening for bleeding	2 (1.2%)	1 (0.7%)	3 (1.9%)	0.63
Blood Units transfusion (mean value)	1.5 ± 2.2	1.9 ± 2.7	2.1 ± 4.4	0.18
FFP Units transfusion (mean value)	0.3 ± 1.4	0.3 ± 1.1	0.5 ± 2.4	0.53
Platelet Units transfusion (mean value)	0.2 ± 0.9	0.2 ± 1.1	0.2 ± 1	0.97

ICU = Intensive Care Unit; LOS = low cardiac output syndrome; IABP = intra-aortic balloon pump; cTnI = cardiac Troponin I peak; AF=atrial fibrillation; CVA = cerebro-vascular accident; FFP = fresh frozen plasma.
